# Preanalytical Quality Evaluation of Citrate Evacuated Blood Collection Tubes—Ultraviolet Molecular Absorption Spectrometry Confronted with Ion Chromatography

**DOI:** 10.3390/molecules28237735

**Published:** 2023-11-23

**Authors:** Nataša Gros, Tjaša Stopar

**Affiliations:** Faculty of Chemistry and Chemical Technology, University of Ljubljana, Večna Pot 113, SI1000 Ljubljana, Slovenia; ts4764@student.uni-lj.si

**Keywords:** blood collection tubes, citrate anticoagulant concentration, direct spectrometric determination, ion exclusion chromatography, ion exchange chromatography, heparin, calcium contamination, lithium contamination, potassium contamination, magnesium contamination

## Abstract

We previously enabled a direct insight into the quality of citrate anticoagulant tubes before their intended use for specimen collection by introducing an easy-to-perform UV spectrometric method for citrate determination on a purified water model. The results revealed differences between the tubes of three producers, Greiner BIO-ONE (A), LT Burnik (B), and BD (C). It became apparent that tubes C contain an additive, which absorbs light in the ultraviolet range and prevents reliable evaluation of citrate anticoagulant concentration with the suggested method. In this research, we re-evaluate the quality of citrate-evacuated blood collection tubes by complementing UV spectrometry with ion chromatography. (1) Comparable results were obtained for tubes B at 220 nm. (2) Citrate concentrations determined with ion chromatography were lower for tubes A and C. Chromatograms reveal additional peaks for both. (3) Influences of heparin on absorption spectra and chromatograms of citrate were studied. Some similarities with the shape of the anticoagulant spectra of tubes A and C were observed, and the lithium heparin peak in chromatograms is close to them, but a confident judgment was not possible. (4) Contamination of anticoagulant solution with potassium, magnesium, and calcium was confirmed for all the brands, and contamination with lithium for B and C.

## 1. Introduction

The preanalytical phase is essential for obtaining reliable test results in haematology and coagulation [1]. Validation of devices before routine use is requested of the laboratory professionals [2]. Results obtained with the new device should be evaluated against the results obtained with already implemented devices. Comparability should be statistically confirmed on human samples. The drawing capacity of the tubes changes over time [3,4]. In addition to other sources of uncertainty, this affects the citrate anticoagulant concentration. If it is too high, it influences the results of coagulation tests [5]. Monitoring the quality change during the tubes’ shelf-life and between batches of human samples is not very convenient and requires ethical considerations.

We recently developed a methodology that provides a complementary insight into evacuated blood collection tube characteristics before specimen collection [6]. It is suggested to control variability in the tubes as a potential source of preanalytical errors. By introducing an easy-to-perform UV spectrometric method for citrate determination on a purified water model, we enabled the evaluation of (1) the accuracy of the anticoagulant amount added into the tubes by a producer, (2) the accuracy of the volume of anticoagulant solution in the tube at the instant of examination, and (3) the anticoagulant concentrations at a draw volume. The results revealed differences between the tubes of three producers, Greiner BIO-ONE (A), LT Burnik (B), and BD (C). It became apparent that tubes C contain an additive, which absorbs light in the ultraviolet range and prevents reliable evaluation of citrate anticoagulant concentration with the suggested method.

Chromatographic methods, by enabling the separation of sample components, overcome the lack of selectivity in direct molecular absorption spectrometry in the ultraviolet and visible range (UV-VIS MAS). High-performance liquid chromatography (HPLC) is a very popular chromatographic technique. Ion chromatography, or more precisely, high-performance ion chromatography (HPIC), even though suitable for clinical and pharmaceutical studies [7], is not so widely used. Instrumentation is not so common. Ion exchange chromatography with suppressed conductometric detection was used for citrate determination in pharmaceutical preparations [8,9]. Ion exclusion chromatography separates species on a different principle; strong electrolytes do not reach the ion exchanger due to the Donnan membrane effect, but carboxylic acids pass through, separate, and can be determined with a suppressed conductometric detector [10,11]. We selected ion exclusion chromatography for citrate determination and ion exchange chromatography for the determination of cationic contaminants.

Tube and stopper materials release substances that can affect the results of blood tests [12,13,14,15,16]. The potential impact of additives and anticoagulants should be well understood [17]. Leeching from the materials should be examined, and additives that are not stated in a declaration should be identified. Data in the literature confirm that heparin absorbs light below 230 nm, less pure forms exhibit a broader absorption peak between 200 and 220 nm and absorb light between 240 and 260 nm [18]. This implies some similarities with what we observed in the anticoagulant spectra of tubes C, which need clarification.

The research objectives of this paper can be expressed in four hypotheses (H1 to H4) and a research question Q1:

**H1.**  *UV-VIS MAS provides relevant insight into the quality of vacuum blood collection tubes for coagulation tests if there are no interfering substances absorbing in the UV range. The hypothesis probably holds for the tubes B, possibly for A, and certainly not for C*.

**H2.**  *Ion exclusion chromatograph is a suitable but not a low-cost alternative for citrate determination. Comparison of the results of citrate anticoagulant concentration determination with UV-VIS MAS can clarify the H1*.

**H3.**  *Ion exclusion chromatography might confirm the presence of additives in the tubes of some producers if additional peaks are going to be observed. It cannot be expected that their identification is necessarily going to be possible within this study.*

**H4.**  *Cationic ion exchange chromatography provides better insight into contamination with cations than atomic absorption spectrometry. Additional contaminants might be identified.*

Q1: What is the influence of heparin on the UV absorption spectra and chromatograms of citrate anticoagulant solutions?

## 2. Results

Assuming that not every reader is equally interested in ion chromatography verification and method performance for citrate determination, which we evaluate in Section 2.1, it should be noted that the evacuated blood collection tubes’ preanalytical quality examination by the two analytical methods follows in Section 2.2. The closing Section 2.3 investigates impurities and additives in evacuated blood collection tubes.

### 2.1. Ion Chromatographic Method for Citrate Determination—Method Performance

Peak-area measurements proved more reliable than citrate-peak heights. Table 1 provides insight into within-day repeatability of retention times, *t*_r_, and peak area at three different concentration levels of citrate model solution. Relative per cent standard deviation (*s*_r_(%)) is 0.16%, and standard deviation (*s*) is 2.5 × 10^4^, which justifies four significant figures in peak-area measurements. All the following results were obtained with peak area measurements. The *s*_r_(%) for *t*_r_ is between 0.1 and 0.3%; three significant figures can be written.

Concentrations of the citrate standard calibration solutions (*x*) extended between 0.25 and 4 mmol/L, equally as for UV-VIS MAS. Under these conditions, peak area (*y*) is not linearly related to the concentration of citrate calibration standards. Data in Table 2 verify the within-laboratory reproducibility of calibration parameters of a polynomial of the second order (*y* = *a* × *x*^2^ + *b* × *x* + *c*) for peak area measurements over a two-and-a-half-month period. Parameters *a*, *b*, and *c* are given together with their standard uncertainties (*s_a_*, *s_b_*, and *s_c_*), a standard error of the estimate (*s_y_*_/*x*_), and a coefficient of determination (*R*^2^).

Table 3 summarises the within-laboratory reproducibility of the mean citrate concentration interpolated from a polynomial of the second order, accompanied by a confidence interval. A model solution was injected in three replications.

The method proved reliable for citrate determination.

### 2.2. Evacuated Blood Collection Tubes for Coagulation Tests—Anticoagulant Concentration Evaluation with the Two Methods

**Figure 1:**
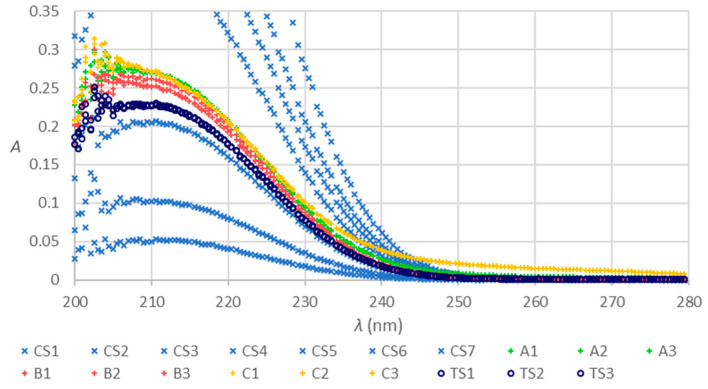
Absorption spectra of trisodium citrate calibration solutions (CS1 to CS7), testing standard solutions derived from the stock standard with the nominal trisodium citrate anticoagulant concentration 10.90 mmol/L as expected for the blood collection tubes after a dilution with a nominal volume (prepared in triplicates TS1 to TS3 and ten-fold diluted), spectra of the composite samples of the blood collection tubes A, B, and C prepared in triplicates by ten-fold dilution, and obtained as described in Section 4.1 and Section 4.3.

**Figure 2:**
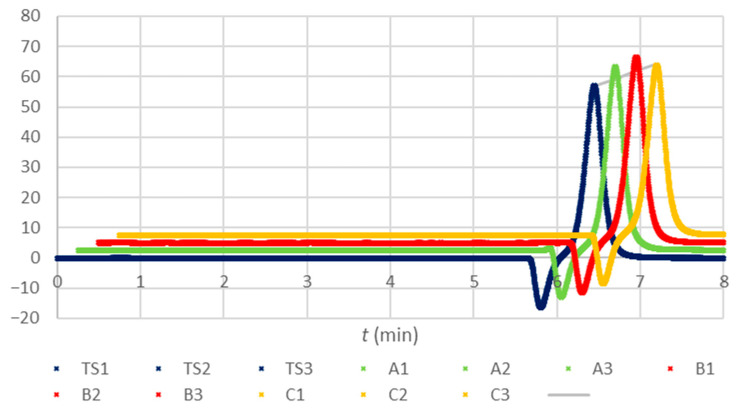
Chromatograms of the testing standard solutions (TS) derived from the stock solution with the nominal trisodium citrate anticoagulant concentration of 10.90 mmol/L as expected for blood collection tubes after a specimen collection (TS1 to TS3, ten-fold diluted), spectra of the composite samples of the blood collection tubes A, B, and C in triplicates, prepared as described in Section 4.1 and Section 4.3. Displacements in x- and y-direction were applied to the chromatograms of different kinds to avoid overlapping and provide better insight. A grey line indicates the peak heights corresponding to the concentration of the testing standard.

The raw data obtained with the two methods are presented in Figure 1 and Figure 2. The abbreviations CS1 to CS7 stand for calibration solutions, and TS1 to TS3 for testing standard solutions prepared by a ten-fold dilution of the stock trisodium citrate solution with the 10.90 mmol/L concentration corresponding to the nominal anticoagulant concentration as expected in the blood collection tubes after a specimen collection. The letters A, B, or C with the numerals from 1 to 3 pertain to composite samples of the corresponding tubes, diluted ten-fold in triplicates. The procedures and tubes are described in Section 4.1 and Section 4.3. Only the tubes within their shelf life were considered (Section 4.1, suffix n in the tube’s name abbreviation). If not stated differently, this applies to all the mentions of the tubes in continuation.

**Figure 3:**
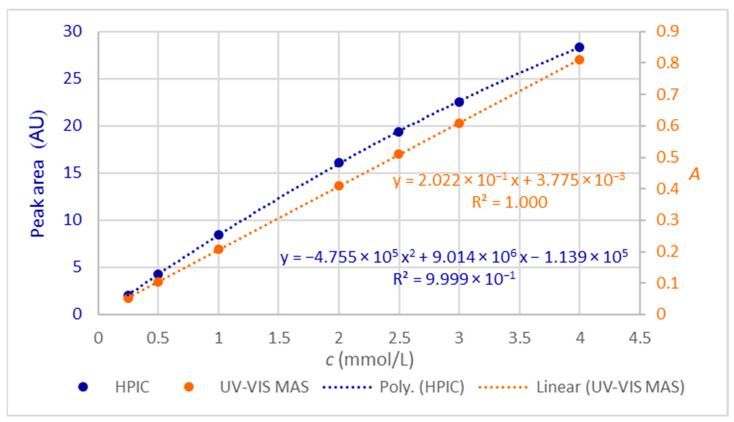
Calibration curves for the calibration standards were prepared as described in Section 4.3 and obtained with HPIC (y-axis to the left, blue) and UV-VIS MAS (y-axis to the right, orange).

The calibration equations for the determination of citrate anticoagulant concentration with high-performance ion chromatography (HPIC) and molecular absorption spectrometry (UV-VIS MAS) are given in Figure 3. The primary y-axis to the left with the peak area in arbitrary units (AU) and data in blue are dedicated to ion chromatography, the secondary y-axis to the right with absorbance (*A*), and the data in orange correspond to molecular absorption spectrometry. For HPIC, the polynomial of the second order was used. The coefficients of determination (*R*^2^) for both methods are very high, exceeding 0.9999 or 99.99%.

The mean concentrations and confidence intervals (2 × *α* = 0.05, *ν* = 2) of citrate determinations in three replicated diluted stock testing standards (TS) with the two methods were 10.82 ± 0.05 mmol/L for HPIC, 11.12 ± 0.14 mmol/L for UV-VIS MAS at 210 nm, and 11.03 ± 0.12 mmol/L for UV-VIS MAS at 220 nm, respectively. The results are given for a stock solution. The determined concentrations were multiplied by a factor of dilution 10. The TS stock concentration of citrate was 10.90 mmol/L. The solution was prepared by weighing and was the same for both methods. The results reflect the combined uncertainty of interpolation and the uncertainty of ten-fold dilution. It was a quality control measure for both methods.

The mean citrate anticoagulant concentrations and their variances determined in the composite samples of tubes A, B, and C with the two methods are summarised in Table 4. We used one-factor ANOVA to test the null hypothesis, stating that the analytical method does not affect the anticoagulant concentration determination in a composite sample obtained from tubes of the same kind. A method choice, either UV molecular absorption spectrometry (UV-VIS MAS) or high-performance ion chromatography (HPIC), was a controlled factor. The examined variable was the concentration of the citrate anticoagulant determined in a composite sample by analysing 10-fold diluted solutions prepared in triplicates. Since the calculated *p*-values were namely 2.40 × 10^–6^ for A, 1.01 × 10^–2^ for B, and 2.01 × 10^–7^, the null hypothesis had to be rejected in all the cases at the 95% confidence level.

For tubes B, with a *p*-value below 0.05 but exceeding 0.01, we tested the null hypotheses also for the results obtained with the UV-VIS MAS at 220 nm instead of 210 nm, where the effects of other interfering substances or cross-contamination are less likely. Under these circumstances and only for this type of tube, the results obtained by the two methods proved comparable (*p* = 0.0743). We next tested the null hypothesis that the UV-MAS at 220 nm does not give the mean anticoagulant concentration, which is significantly higher than the mean concentration obtained with HPIC for the tubes B. Both the *t*-test assuming unequal variances (*t* = 2.40 < *t*_critical_ = 2.92, *ν* = 2, *α* = 0.05) and the paired two samples for means *t*-test (*t* = 2.23 < *t*_critical_ = 2.92, *ν* = 2, *α* = 0.05) confirmed the validity of the null hypothesis. The mean anticoagulant concentration determined for tubes B with the UV-VIS MAS at 220 nm is not significantly higher if compared with HPIC.

### 2.3. Evacuated Blood Collection Tubes for Coagulation Tests—Impurities and Additives Examination

#### 2.3.1. Cations as Contaminants Examined with Cationic Ion Chromatography

**Figure 4:**
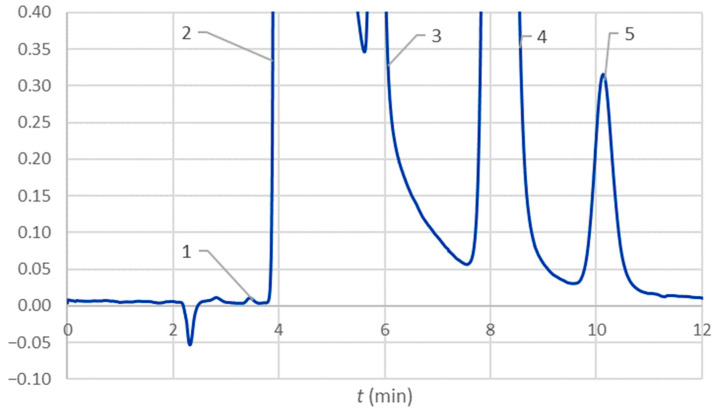
Chromatogram of a composite sample of tube B_1.8 obtained with the cationic ion exchange chromatography, the peak numbers are 1 for lithium, 2 for sodium, 3 for potassium, 4 for magnesium, and 5 for calcium.

The tubes A_1.8, B_1.8, and B_1.8, which were previously examined with atomic absorption spectrometry for the presence of cationic contaminants [6], were re-evaluated with HPIC. It should be noted that this was performed after the expiration dates of the tubes for the sake of comparison. An example of a chromatogram of the composite sample prepared from tube B_1.8 (expiration date 31.12.2021) is presented in Figure 4. The well-expressed peak of potassium—3, which is superimposed on to the tail of the widest peak of sodium—2, and the following peaks of magnesium—4 and calcium—5 were observed for the tubes of all three types. A small peak that precedes the peak of sodium corresponds to lithium—1. In addition to the example presented for tubes B, lithium was also observed for tubes C but not for A. Lithium and potassium concentrations determined in composite samples are given in Table 5. Limits of detection (LoD) estimated from *b*, a *s_y/x_*, as suggested in the literature [19], were 0.08 for lithium, 6 for potassium, 5 for magnesium, and 0.6 for calcium, respectively, expressed in µmol/L.

#### 2.3.2. Absorption Spectra and Chromatograms of Citrate Influenced by Heparin

**Figure 5:**
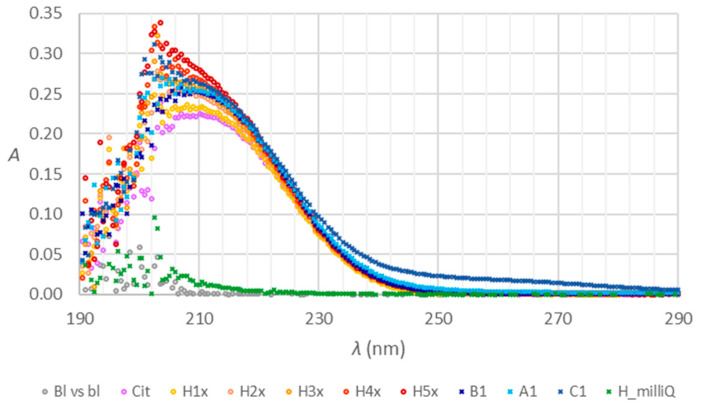
Absorption spectra of heparin (H_milliQ), citrate (Cit), heparin and citrate mixtures (H1x to H5x), and the composite samples of the tubes A, B, and C prepared and evaluated as explained in Section 4.4.

Figure 5 presents the influences of heparin on absorption spectra under the experimental conditions as used in the spectrometric method for citrate determination. The Bl vs bl stands for 126 mol/L HCl blank measured against itself, Cit is trisodium citrate model solution, H1x to H5x is citrate contaminated with an increasing amount of heparin, H_milliQ stands for heparin in Milli-Q water. Other symbols are as previously described. The preparation of solutions is explained in Section 4.4.

**Figure 6:**
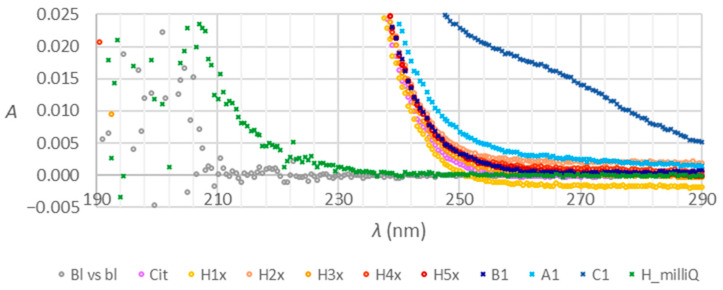
Absorption spectra of heparin (H_milliQ), citrate (Cit), heparin and citrate mixtures (H1x to H5x), and the composite samples of the tubes A, B, and C prepared and evaluated as explained in Section 4.4., a closer look.

Figure 6 provides a closer look at the spectra from Figure 5 in the lower absorbance range.

**Figure 7:**
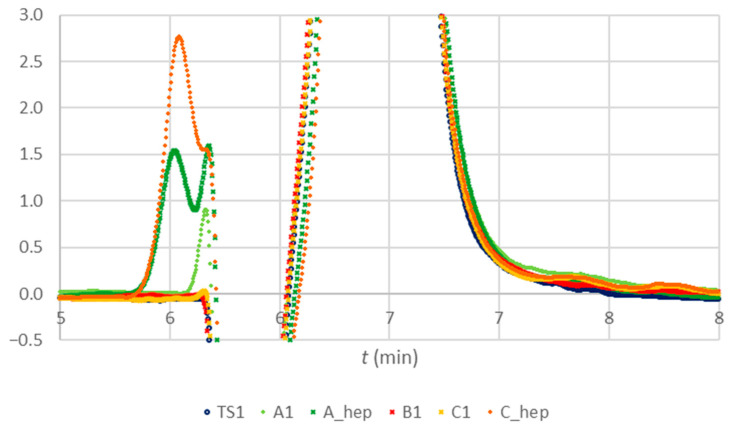
Chromatograms of a testing standard solution (TS1), the first replication of ten-fold diluted composite samples (A1, B1, and C1), together with diluted samples A and C spiked with heparin (A_hep and C_hep), zoomed-in around the water deep and the base of the citrate peak.

In Figure 7, the zoomed-in section of the chromatograms of TS1, A1, B1, and C1 (Figure 2) are confronted with the chromatograms obtained for the composite samples spiked with heparin (A_hep and C_hep). As a source of heparin, BD blood collection tubes were used; a full specification is given in Section 4.4.

## 3. Discussion

### 3.1. Ion Chromatographic Method for Citrate Determination—Method Performance

To exclude additional sources of uncertainty due to different dilution steps, we decided to use the same calibration range for the determination of citrate with HPIC as previously with UV-VIS MAS, which extended from 0.25 to 4 mmol/L. The objectives of DeBorba [8] and Jenke [9], who used ion exchange chromatography for citrate and phosphate determination, differed from ours; no inorganic ions were expected in our case. Ion exchange column AS11 has 600 times lower capacity per column than ion exclusion column ICE-AS1. Both authors had to use a 10-µL injection loop, which is unfavourable, even though the calibration ranges were below the range relevant to us.

A preliminary experiment confirmed that peak-area measurement is more reliable than peak-height measurement; therefore, the former was used in continuation. A test of within-day repeatability of retention times and peak area measurements at the edges and in the middle of the calibration range confirmed the *s*_r_(%) below 0.35% for all the cases (Table 1).

Seven-point calibration performed during nearly two and a half months proved that a polynomial of the second order should be used to describe the relation between citrate concentration and peak area measurement. Reproducibility of the parameters of the polynomial, their standard uncertainty, standard error of estimate, and coefficient of determination, *R*^2^, are shown in Table 2. *R*^2^, by having the lowest value of 0.9974 and the highest value of 0.9999, observed in 6 out of 12 cases, confirms that the selected polynomial model well describes experimental data.

Results of within-laboratory reproducibility of interpolation of mean citrate concentrations of a model solution from a polynomial of the second order and the confidence intervals agree well between days (Table 3). The model solution was freshly prepared on each testing day and injected in triplicates. The determined concentrations obtained during more than two months extended between 2.004 ± 0.004 mmol/L and 2.07 ± 0.01 mmol/L. The HPIC method proved reliable for citrate determination. The first part of hypothesis H2, claiming that HPIC is a suitable alternative for citrate determination, even though not a low-cost one, is confirmed.

### 3.2. Evacuated Blood Collection Tubes for Coagulation Tests—Anticoagulant Concentration Evaluation with the Two Methods

Absorption spectra of the composite samples of tubes A, B, and C (Figure 1) are much more similar in absorbance at the wavelength of absorption maximum than the previous spectra [6], which were obtained after the addition of the nominal volume of Milli-Q water directly into a single tube. A composite sample eliminates the effect of the potentially noncompliant volume of citrate stock anticoagulant solution in a tube. The confirmation that tubes B lose water over time [6] additionally contributes to the difference between the results of the two experiments. Regarding the shape of the spectra, similar characteristics were confirmed, as observed previously. The spectra of tubes C exhibit different shapes in the range of absorption maximum and also between 230 and 280 nm. Obviously, some other substances absorbing UV light are present. Hypothesis H1 is relevant concerning the new series of tubes and needs clarification.

In the chromatograms of composite samples (Figure 2), no peaks that could have interfered with the citrate peak area integration were observed. The HPIC method’s fitness for purpose can be assumed.

Coefficients of determination of the calibration functions (Figure 3), namely polynomial of the second order for HPIC and linear regression equation for UV-VIS MAS, confirm that the models explain more than 99.99% of the experimentally obtained data. The polynomial equation matches well the data in Table 2. The linear equation is compliant with the previously reported data [6].

**Figure 8:**
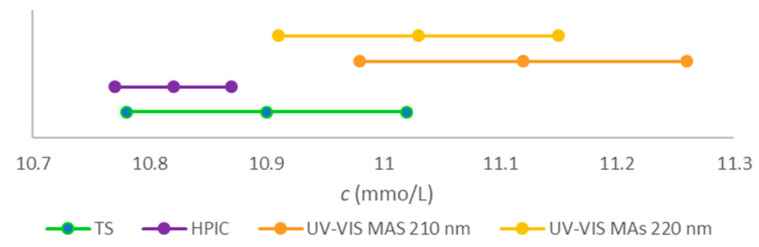
The mean citrate concentration of the testing standard solutions (TS1 to TS3) with the associated expanded standard uncertainty (*U*) with the coverage factor *k* = 2 (green line), compared with the mean citrate concentrations with the confidence intervals obtained with HPIC (violet line), UV-VIS MAS at 210 nm (yellow line), and UV-VIS MAS at 220 nm (orange line).

The testing standard, TS, which had a quality control role in both methods, was prepared in triplicates from the stock citrate standard solution by ten-fold dilution either with Milli-Q water for HPIC or with 140 mmol/L solution of HCl for UV-VIS MAS. The stock was prepared by weighing the chemical, dissolution, and dilution in a volumetric flask with Milli-Q water. The standard uncertainty of the concentration might be considered negligible if compared with the uncertainty contribution caused by dilution, during which 1 mL of the stock was added to 9 mL of a diluent. The resulting expanded standard uncertainty *U*, with a coverage factor 2, was estimated to be 0.12 mmol/L at the 10.90 mmol/L concentration. The range is presented in Figure 8 in green and compared with the results obtained with HPIC and UV-VIS MAS at two different wavelengths. All the results are shown with confidence intervals obtained from the analyses of the TS1, TS2, and TS3 solutions carried out, as explained in Section 2.2. Even though the ranges of the two methods overlap with the TS interval, it is observed that the range of the UV-VIS MAS at 210 nm is shifted towards higher values. The TS solutions were analysed with both methods at the end of the series, immediately after the composite sample C, in which other UV light-absorbing substances in addition to citrate are present. The cross-contamination in UV-VIS MAS cannot be excluded. If this was the case, it is reasonable to expect that the influence is lower at 220 nm. We suggest that the testing standards are analysed not only at the end of the series, as it was in our case, but also immediately after the calibration standards.

One-factor ANOVA with a method choice as the controlled factor and citrate anticoagulant concentration as the response variable confirmed the differences between the two methods for tubes A, B, and C if the absorbance was measured at 210 nm (Table 4). As already discussed, concerning TS, interfering effects at this wavelength are more likely than at higher wavelengths. If the ANOVA test was repeated by considering the results obtained with the UV-VIS MAS at 220 nm, the two methods proved comparable. Both the *t*-test assuming unequal variances and the paired two samples for means *t*-test confirmed that the mean anticoagulant concentration determined for tubes B with the UV-VIS MAS at 220 nm is not significantly higher if compared with HPIC. The results imply that tubes B do not contain interferants influencing the citrate anticoagulant concentration determination with UV-VIS MAS if absorbances are measured at the wavelength at which cross-contamination is less likely, namely at 220 nm.

We confirmed hypothesis H1, claiming that UV-VIS MAS provides relevant insight into the quality of vacuum blood collection tubes for coagulation tests if there are no interfering substances absorbing in the UV range. As we assumed, the hypothesis holds for tubes B. The anticoagulant solution of tubes A and C contain interfering substances that absorb UV light and affect the results obtained with the UV-VIS MAS. For tubes C, this was already clearly expressed in Figure 1. We also confirmed the second part of hypothesis H2, that comparison of the results of citrate anticoagulant concentration determination with UV-VIS MAS with the results obtained with HPIC can clarify the H1.

### 3.3. Evacuated Blood Collection Tubes for Coagulation Tests—Impurities and Additives Examination

#### 3.3.1. Cations as Contaminants Examined with Cationic Ion Chromatography

HPIC proved more potent for the determination of cationic contaminates than atomic absorption spectrometry (AAS) used previously [6]. This is not surprising due to the presence of trisodium citrate in high concentration. As Figure 4 and Table 5 demonstrate, potassium, magnesium, and calcium were detected in the tubes of all three producers. Stoppers can be considered a source of magnesium and calcium [12]. Concentrations of calcium are lower than those of magnesium, around 5 µmol/L. We also detected lithium in tubes B and C but in very low concentrations (Table 5). A possible source might be cross-contamination with lithium heparin at the production line.

The concentration of potassium was only determinable in tubes C with AAS [6]. With HPIC, we confirmed contamination with potassium also for tubes A and B, but at a lower level (Table 5). We tested the null hypothesis that the results obtained for tubes C with AAS and HPIC do not differ significantly. Comparison of the two means with a *t*-test assuming comparable variances, previously confirmed with an F-test, proved that the null hypotheses cannot be rejected at the significance level 0.05 (*p* = 0.031).

The results confirmed the hypothesis H4.

#### 3.3.2. Absorption Spectra and Chromatograms of Citrate, Influenced by Heparin

To answer question Q1, we examined how heparin as a potential contaminant or additive affects the shape of citrate anticoagulant spectra. Pure heparin is expected to absorb light below 230 nm, and absorbance increases toward 190 nm. Contaminated heparin has a broader peak between 200 and 220 nm and absorbs light between 240 and 260 nm [18]. The green trace in Figure 6 shows that heparin absorbance increases exponentially below 240 nm. Consequently, the spectra of citrate solutions contaminated with heparin (H1x to H5x in Figure 5) exhibit a rising tendency below 220 nm. Contrastingly, the spectrum of citrate (Cit in Figure 5) has a flat peak top with an absorbance maximum of 210 nm. The trace of tube C also has a rising part below 220 nm. The same is slightly expressed in the spectrum of tubes A but not in B. Tubes C have a distinct shape of the spectrum above 240 nm (Figure 6). A similar tendency is observed for tubes A but to a lower extent. Heparin influence does not fully explain the shape of the spectra of tubes A and C. There are similarities between our observations and the literature data, but additional confirmation is necessary. Heparin, if present, can affect the results of coagulation tests [20,21,22].

A closer look at the chromatograms (Figure 7) of testing citrate anticoagulant standard and composite samples of tubes A, B, and C revealed a small peak (green trace, tube A) and a very small peak (yellow trace, tube C) at the front edge of the water deep at 5.66 min. A chromatogram of a composite sample A, spiked with lithium heparin (A-hep), demonstrates a double peak with an associated peak at 5.52 min. A chromatogram of a composite sample C, spiked with lithium heparin (C-hep), shows a peak at 5.54 min, followed by an overlapping peak at 5.66. With ion exclusion chromatography, we confirmed additional peaks, as hypothesis H3 assumed.

Ion exchange chromatography used for heparin determination was reported in the literature; ion exclusion chromatography was only mentioned as a possible alternative [23,24]. Some additional experiments would have been needed to fully understand the observations and to identify additives.

#### 3.3.3. Implications and Limitations

We determined citrate concentration in composite samples, which we prepared from ten tubes of the same kind to avoid the effect of potentially inaccurate volume of stock anticoagulant solution in a tube at the point of examination [6]. By doing so, we were in control of the final solution volume. Drying the anticoagulant in individual tubes at 50 °C under the stream of nitrogen did not lead to a desirable outcome. Instead of being entirely solid, the residual, in some cases, turned into a gel-like substance. The composite sample approach was, for us, the best possible, but it can lead to under-estimation of citrate concertation if the transfer of anticoagulant is not entirely quantitative.

## 4. Materials and Methods

### 4.1. Trisodium Citrate, Purified Water, Evacuated Blood Collection Tubes, and Composite Samples

All the chemicals were of the analytical reagent grade. Trisodium citrate dihydrate C_6_H_5_Na_3_O_7_ × 2H_2_O (*M* = 294.10 g/mol, *w* ≥ 0.99), CAS: 6132-04-3, Sigma-Aldrich, St. Louis, MO, USA, was used to prepare calibration standards and model solutions for UV-MAS and HPIC. More details on other chemicals are given concerning dedicated procedures.

Deionised water, additionally purified through the Milli-Q system (Millipore, Billerica, MA, USA), was used to prepare all solutions—Milli-Q water in continuation.

The evacuated blood collection tubes were obtained from the Slovene local dealers. The letters assigned to particular tubes were the same as previously. The letter A stands for Vacuette^®^, Greiner BIO-ONE; B for Vacutube, LT Burnik d.o.o., Skaručna, Slovenia; and C for BD Vacutainer^®^. More details are summarised in Table 6. The letter n was added to the end of the abbreviation of the tubes which were not yet involved in the preceding study and were evaluated within their shelf life.

A composite sample of tubes A, B, or C was prepared from ten tubes of the same kind and the same lot. The content of each tube was quantitatively transferred into an A-class 20 mL volumetric flask with three successive additions of 455 µL of Milli-Q water. After each addition, the tube was plugged and inverted several times, and the content was transferred into the flask through a funnel. The potential residuals of citrate were afterwards washed out of the funnel into a volumetric flask, and the volume of solution was made up to the mark with Milli-Q water.

### 4.2. Ion Chromatography

The DX-500 Ion Chromatograph (Dionex Corporation, Sunnyvale, CA, USA) consisting of the GP-40 Gradient Pump (Sunnyvale, CA, USA) and the ED-40 Electrochemical detector (Sunnyvale, CA, USA) with a conductometric cell was used.

#### 4.2.1. Determination of Citrate with Ion Exclusion Chromatography

For ion exclusion chromatography, the system consisted of a 50 µL injection loop, IonPac ICE-AS1 II (9 × 250 mm) column, and AMMS-ICE II suppressor used in open mode of operation with chemical regeneration. The regenerator solution with the concentration 5 mmol/L was prepared from tetra-n-butylammonium hydroxide (TBAOH, [CH_3_(CH_2_)_3_]_4_NOH, *M* = 259.478 g/mol, 0.395 ≤ *w* ≤ 0.410, *ρ* = 0.995 g/cm^3^ at 20 °C, CAS: 2052-49-5, Sigma Aldrich, Steinheim, Germany), the flow rate was 5 mL/min. The eluent was 1 mmol/L solution of heptafluorobutyric acid (HFBA, C_4_HF_7_O_2_, *M* = 214.04 g/mol, *w* > 0.990, *ρ* = 1.645 g/cm^3^ at 25 °C, CAS: 375-22-4, Sigma Aldrich, Buchs, Switzerland). The flow rate was 0.8 mL/min.

#### 4.2.2. Determination of Cations with Ion Exchange Chromatography

For determination of cations with ion exchange chromatography, a 50 µL injection loop, IonPac CS12A (4 × 250 mm) column, IonPac CG12A (4 × 250 mm) guard column, and CSRS 300 (4 mm) suppressor in closed mode of operation were used. The current was set to 50 mA. The eluent was 22 mmol/L methanesulfonic acid, CH_3_SO_3_H (*M* = 96.11 g/mol, *w* ≥ 0.990, *ρ* = 1.481 g/cm^3^ at 25 °C, CAS: 75-75-2, Sigma Aldrich, Steinheim, Germany), flow rate was 1 mL/min.

Potassium chloride, KCl ((*M* = 74.55 g/mol, 0.990 ≤ *w* ≤ 1.005), Fluka, Seelze, Germany) was dried at 300 °C for one hour. Five potassium ion calibration solutions, prepared from the chemical, had concentrations in the range between 0.75 and 3.75 mg/L.

Lithium standard solution, *γ*(Li^+^) 1000 mg/L (traceable to SRM from NIST, LiNO_3_ in HNO_3_ 0.5 mol/L, *w*(Li^+^) ± *U* = (981 ± 5) mg/kg, *ρ* = 1.017 g/cm^3^, Merck KGaA, Darmstadt, Germany) was used to prepare intermediate standard solution with concentration 0.5 mg/L. Five lithium ion calibration solutions with concentrations between 1 and 3 µg/L were prepared from it.

### 4.3. Comparison of Molecular Absorption Spectrometry and Ion Chromatography for Citrate Determination in Evacuated Blood Collection Tubes

A set of seven stock calibration solutions with citrate concentrations from 2.5 to 40 mmol/L was prepared in volumetric flasks by weighing the trisodium citrate dihydrate. No mass was lower than 140 mg. A diluent was Milli-Q water. A 10.90 mmol/L citrate testing standard solution was prepared similarly. The composite samples of tubes A, B, or C were prepared, as described in Section 4.1.

For ion chromatography or molecular absorption spectrometry, all the solutions were 10-fold diluted by adding 1 mL of the respected stock solution to 9 mL of diluent. In the first case, the diluent was purified water, and in the second, 140 mmol/L HCl solution, prepared from the 37% HCl (CAS: 7647-01-0, *ρ* = 1.19 g/mL, Honeywell, Seelze, Germany).

The diluted composite samples (A1 to A3, B1 to B3, and C1 to C3) and testing standard (TS1 to TS3) were prepared in triplicates from the stock solutions. Spectrometric measurements were performed using Varian Cary 50 UV-Vis spectrometer, Agilent Technologies, Santa Clara, CA, USA, ZDA in 10 mm QS high Precision Cell (Hellma Analytics; Müllheim, Germany), against 126 mmol/L HCl solution. Absorbance was measured in the wavelength range between 190 nm and 350 nm.

### 4.4. Heparin Influence on Absorption Spectra

BD Vacutainer LH (Lithium Heparin) 34 I.U. Plus Blood collection tubes, 2.0-mL (2024 03), were used as a source of heparin (BD, Plymouth, UK).

In the first of the LH tubes, 2 mL of Milli-Q water was added. 10.90 mmol/L trisodium citrate was used as a model solution, and 2 mL was introduced into each of the five LH tubes. The content was well-mixed. The first tube was kept as it was. The content of every other tube was transferred into an additional LH tube. A procedure was repeated so that we could obtain a series of the citrate model solutions, which were in contact with the lithium heparin from one to five LH tubes (H1x to H5x). In tubes A_1.8_n, B_1.8_n, or C_1.8_n, the nominal volume of the Milli-Q water was measured, and the content was well-mixed. For spectrometric measurement, a citrate model solution and all other solutions were 10-fold diluted with 140 mmol/L HCl solution. Varian Cary 50 UV-Vis spectrometer and QS high Precision Cell (Hellma Analytics), with a light path of 10 mm, were used to obtain the absorption spectra in the wavelength range from 190 to 400 nm. The measurements were performed against a 126 mmol/L HCl blank solution.

## 5. Conclusions

We confirmed that UV-VIS MAS provides relevant insight into the quality of vacuum blood collection tubes for coagulation tests if there are no interfering substances absorbing in the UV range. This holds for tubes B if we measure absorbance at 220 nm, where interfering effects and cross-contamination are less expressed. UV-VIS MAS overestimated the citrate concentration of tubes A and C due to the presence of other interfering substances.

We proved that the ion exclusion chromatograph is a suitable but not a low-cost alternative for citrate determination. Comparison of the results of citrate anticoagulant concentration determination with UV-VIS MAS clarified hypothesis H1.

Ion exclusion chromatography confirmed the presence of additives in tubes A and C. Additional peaks were observed. Their identification was not possible within this study.

Cationic ion exchange chromatography provided better insight into contamination with cations than atomic absorption spectrometry. Lithium and calcium as additional contaminants were identified.

We examined the influence of heparin on the UV absorption spectra and chromatograms of citrate anticoagulant solutions. Some similarities were observed, but further clarifications are necessary.

## Figures and Tables

**Table 1:** Within-day repeatability (*n* = 5) of retention times (*t*_r_) and peak area measurements for trisodium citrate model solution at three concentration levels: 0.25 mmol/L, 2 mmol/L, and 4 mmol/L.

Repetition	1.	2.	3.	4.	5.	$\bar{x}$	*s*	*s*_r_ (%)
*c* (mmol/L)	*t*_r_ (min)							
0.25	6.40	6.43	6.43	6.39	6.39	6.41	0.02	0.3
2	6.52	6.54	6.53	6.54	6.53	6.53	0.01	0.1
4	6.59	6.56	6.59	6.60	6.60	6.59	0.02	0.2
*c* (mmol/L)	Peak area (AU)							
0.25	2.199 × 10^6^	2.201 × 10^6^	2.206 × 10^6^	2.187 × 10^6^	2.195 × 10^6^	2.198 × 10^6^	7.0 × 10^3^	0.32
2	1.601 × 10^7^	1.597 × 10^7^	1.595 × 10^7^	1.600 × 10^7^	1.592 × 10^7^	1.597 × 10^7^	3.8 × 10^4^	0.24
4	2.780 × 10^7^	2.768 × 10^7^	2.762 × 10^7^	2.771 × 10^7^	2.774 × 10^7^	2.771 × 10^7^	6.6 × 10^4^	0.24

**Table 2:** Within-laboratory reproducibility of parameters of the polynomial calibration function of the second order (*y* = *a* × *x*^2^ + *b* × *x* + *c*) and their standard uncertainties (*s_a_*, *s_b_*, and *s_c_*), standard error of estimate (*s_y_*_/*x*_) and coefficient of determination (*R*^2^) for peak-area measurements (*y*) in dependence of concentration (*x*) obtained by LINEST function in MS Excel (Microsoft Office Professional Plus 2019).

Date	*a*	*s_a_*	*b*	*s_b_*	*c*	*s_c_*	*s_y/x_*	*R* ^2^
13 March 2023	−4.98 × 10^5^	3.50 × 10^4^	8.85 × 10^6^	1.47 × 10^5^	8.89 × 10^4^	1.22 × 10^5^	1.28 × 10^5^	0.9999
14 March 2023	−4.86 × 10^5^	4.21 × 10^4^	8.85 × 10^6^	1.77 × 10^5^	9.18 × 10^4^	1.46 × 10^5^	1.54 × 10^5^	0.9998
15 March 2023	−5.03 × 10^5^	3.41 × 10^4^	9.11 × 10^6^	1.43 × 10^5^	5.91 × 10^4^	1.19 × 10^5^	1.25 × 10^5^	0.9999
16 March 2023	−4.84 × 10^5^	4.36 × 10^4^	9.10 × 10^6^	1.83 × 10^5^	1.16 × 10^5^	1.52 × 10^5^	1.60 × 10^5^	0.9998
20 March 2023	−4.87 × 10^5^	2.74 × 10^4^	9.04 × 10^6^	1.15 × 10^5^	4.15 × 10^3^	9.54 × 10^4^	1.00 × 10^5^	0.9999
28 March 2023	−4.38 × 10^5^	2.86 × 10^4^	8.16 × 10^6^	1.20 × 10^5^	2.72 × 10^5^	9.96 × 10^4^	1.05 × 10^5^	0.9999
29 March 2023	−4.79 × 10^5^	3.63 × 10^4^	8.26 × 10^6^	1.52 × 10^5^	2.62 × 10^5^	1.26 × 10^5^	1.33 × 10^5^	0.9998
30 March 2023	−4.48 × 10^5^	9.58 × 10^4^	8.11 × 10^6^	4.02 × 10^5^	3.53 × 10^5^	3.33 × 10^5^	3.50 × 10^5^	0.9989
3 April 2023	−3.18 × 10^5^	1.46 × 10^5^	7.51 × 10^6^	6.12 × 10^5^	5.96 × 10^5^	5.07 × 10^5^	5.33 × 10^5^	0.9974
22 May 2023	−3.93 × 10^5^	9.80 × 10^4^	8.27 × 10^6^	4.11 × 10^5^	1.39 × 10^5^	3.41 × 10^5^	3.58 × 10^5^	0.9990
23 May 2023	−4.92 × 10^5^	3.37 × 10^4^	8.84 × 10^6^	1.41 × 10^5^	−3.51 × 10^4^	1.17 × 10^5^	1.23 × 10^5^	0.9999
24 May 2023	−5.44 × 10^5^	4.02 × 10^4^	9.17 × 10^6^	1.69 × 10^5^	−3.86 × 10^4^	1.40 × 10^5^	1.47 × 10^5^	0.9998
25 May 2023	−5.03 × 10^5^	3.54 × 10^4^	8.95 × 10^6^	1.49 × 10^5^	−4.35 × 10^4^	1.23 × 10^5^	1.30 × 10^5^	0.9999

**Table 3:** Within-laboratory between-day reproducibility of citrate concentration interpolation from a polynomial of the second order, the determined mean concentration of citrate model solution is given together with its confidence interval (2 × *α* = 0.05, *ν* = 2).

Date	16 March 2023	20 March 2023	28 March 2023	30 March 2023	3 April 2023	22 May 2023	23 May 2023	24 May 2023	25 May 2023
$\bar{c}$ (mmol/L)	2.007	2.004	2.005	2.03	2.07	2.05	2.014	2.004	2.019
$\pm t\times s/\sqrt{n}$	0.004	0.004	0.005	0.02	0.01	0.03	0.004	0.008	0.005

**Table 4:** Results of the ANOVA test for the anticoagulant concentration determined in the composite samples prepared from the tubes of all three producers, the asterisk at a hanging indent indicates that the between-groups difference proved statistically significant.

**A_1.8_n**	**Count**	**Sum**	**Average**	**Variance**	**Differences**				
UV-VIS MAS	3	39.51	13.17	4.49 × 10^–3^	*				
HPIC	3	34.85	11.62	9.80 × 10^–5^		*			
**B_1.8_n**	**Count**	**Sum**	**Average**	**Variance**	**Differences**				
UV-VIS MAS	3	37.32	12.44	5.73 × 10^–2^	*				
HPIC	3	35.40	11.80	4.31 × 10^–4^		*			
**B_1.8_n (220 nm)**	**Count**	**Sum**	**Average**	**Variance**	**Differences**				
UV-VIS	3	36.48	12.16	6.63 × 10^–2^	*				
HPIC	3	35.40	11.80	4.31 × 10^–4^	*				
**C_1.8_n**	**Count**	**Sum**	**Average**	**Variance**	**Differences**				
UV-VIS	3	39.71	13.24	4.289 × 10^–3^	*				
HPIC	3	31.96	10.65	2.05 × 10^–4^		*			

**Table 5:** Lithium and potassium concentrations and their standard deviations (*s*) determined in composite samples prepared from the blood collection tubes A, B, or C.

Tubes	Expiration Date	*c*(Li^+^) (μmol/L)	*s* (*n* = 3)	*c*(K^+^) (μmol/L)	*s* (*n* = 3)
A_1.8_9.4.	9 April 2022 **	/	/	24	±1
B_1.8	31 December 2021 **	0.15	±0.07	47.3	±0.9
C_1.8	30 September 2021 **	0.25	±0.01	127	±6

** After the expiration date for the sake of comparison with the previously obtained data [6].

**Table 6:** Characteristics of the examined evacuated citrate or buffered citrate blood collection tubes.

Abbreviation	Anticoagulant *c* (mmol/L)	Expiration Date	Draw Volume (mL)
A_1.8_n	109	4 July 2024	1.8
B_1.8_n	109	31 January 2024	1.8
C_1.8_n	109 *	31 December 2023	1.8
A_1.8 **	109	9 April 2022	1.8
B_1.8 **	109	31 December 2021	1.8
C_1.8 **	109 *	30 September 2021	1.8

* Buffered trisodium citrate. ** Included because they were involved in the previous study [6].

## Data Availability

Data is contained within the article.
